# Bleeding complications in patients on warfarin undergoing joint injection/aspiration: systematic review and meta-analysis

**DOI:** 10.1007/s00296-022-05232-y

**Published:** 2022-11-02

**Authors:** M. Y. Tarar, R. A. Malik, C. P. Charalambous

**Affiliations:** 1 Blackpool Teaching Hospitals NHS Trust, Blackpool, UK; 2 Weill Cornell Medicine-Qatar, Doha, Qatar; 3 University of Manchester, Manchester, UK; 4 School of Medicine, University of Central, Lancashire, UK

**Keywords:** Intra-articular injection, Arthrocentesis, Warfarin, Bleeding

## Abstract

Steroid injections in joints are commonly administered for the management of inflammatory or degenerative conditions. There is substantial controversy as to whether to continue warfarin when undertaking joint injection or aspiration. To assess the rate of bleeding complications in patients on warfarin undergoing joint injection/aspiration. Systematic review and meta-analysis. A literature search of 3 online databases was conducted by 2 reviewers using the Cochrane methodology for systematic reviews. Eligibility criteria were any study that reported bleeding complication rates in adult patients on warfarin undergoing a joint injection/aspiration whilst taking warfarin anticoagulation. Studies reporting on less than 5 patients were excluded. Meta-analysis was conducted using a random effects model. The search of databases resulted in a total of 1547 articles. After screening, 8 articles were deemed suitable for inclusion in the analysis, involving 871 injection/aspiration procedures. There were only 5 reported cases of bleeding. On meta-analysis the estimated bleeding complication rate was 1.5% (95% CI 0.5–4.5%). This meta-analysis shows that it is safe to perform joint injection and aspiration in patients on warfarin without routine prior testing of INR. Level of evidence: Level 4.

## Introduction

Steroid injections in joints are commonly administered for the management of inflammatory or degenerative conditions. [1, 2] There is a substantial body of data to support a benefit of intra-articular hyaluronic acid, glucocorticoids, platelet-rich plasma and mesenchymal stem cells in knee and hip osteoarthritis and shoulder capsulitis [3]. Indeed, of 545 consultations at an urban community general practice, 115 (21.1%) involved a musculoskeletal presentation, of these 17.4% involved the knee in which steroid injections were administered in 33% [1].

It is estimated that in the UK up to 1.25 million people are currently prescribed oral anticoagulants [4], with 6% in the 80–84 year age group taking warfarin [5] for common indications like atrial fibrillation, deep venous thrombosis, or pulmonary embolism. Many of these elderly patients on warfarin will present with musculoskeletal complaints necessitating intra-articular injection or aspiration.

There is substantial controversy and no consensus on continuing or stopping warfarin prior to joint injection or aspiration to avoid the risk of bruising or hemarthrosis. Indeed, whilst some have recommended stopping and reversing the effect of warfarin [6, 7], others have argued that stopping oral anticoagulants may increase the risk of life-threatening thromboembolic events [8]. Indeed, EULAR (European alliance of associations for Rheumatology) have recently developed the first evidence and expert opinion-based recommendations to guide health professionals using intra-articular therapy (IAT) and concluded that the risk of peri-procedural bleeding was low for patients on anti-thrombotic drugs [9].

A robust evaluation of the safety of joint injections or aspirations in patients on warfarin may inform clinician-patient discussion as part of the shared decision making and consent process. The aim of this study was to establish through a systematic review and meta-analysis the rate of bleeding complications in patients on warfarin undergoing intra-articular injection or aspiration.

## Materials and methods

A literature search of MEDLINE (1946 to present), EMBASE (1974 to present) and Cochrane CENTRAL (1988 to present) databases was conducted using combination of the key words “Injection”, “shoulder”, “elbow”, “wrist”, “hand”, “hip”, “knee”, “ankle”, “foot”, “joint”, “intra-articular”, “aspiration”, “arthrocentesis”, “warfarin” and “anticoagulation) in April 2022 for articles published in any language with no publication year limit. Searches were performed with specific keywords rather than medical subject headings to avoid missing any relevant studies.

### Eligibility criteria for inclusion

Study design: Any study design, including randomized controlled studies, prospective cohort studies, retrospective cohort studies, case control studies and case series including more than 5 patients. Case reports and reviews were excluded.

Population: Patients older than 18 years taking warfarin.

Intervention: Joint injection or aspiration.

Outcomes: Bleeding complications.

Data were extracted using an electronic standardized proforma.

Two reviewers (YT, CPC) independently screened the titles and abstracts of all identified studies for inclusion and duplicates were removed. Full texts of eligible studies were retrieved and reviewed. The reference lists of all included articles were searched for any additional articles not identified through the database search. Disagreements for inclusion were discussed between reviewers and if not resolved with one of the senior authors. Additional data was requested from the authors when deemed necessary and added into the data pool. The Preferred Reporting Items for Systematic Review and Meta-Analyses (PRISMA) methodology was used [10]. The design methodology of each study was determined using the guidelines described by Mathes and Pieper. [11] The Cochrane Risk of Bias Tool was used to assess the risk of bias in the included RCTs and the Methodological Index for Non-Randomized Studies (MINORS criteria) for non-randomized studies (MINORS) tool for assessment of bias in observational studies. The Grading of Recommendations, Assessment, Development, and Evaluation (GRADE) approach was used to assess the quality of evidence of this review [12]. The protocol was not registered or published prospectively.

### Statistical analysis

An initial descriptive analysis of the studies was performed, presenting study characteristics, populations, and outcomes. Meta-analysis was conducted using a random-effects model, due to the inherent heterogeneity encountered in clinical studies. Estimated rates of bleeding complications and 95% confidence intervals (CIs) were calculated and reported. Heterogeneity was assessed using tau^2^, *I*^2^, *Q* and *p* values. Small study effect and publication bias was assessed visually using a funnel plot. Data were analyzed with Comprehensive Metanalysis version 2 (Biostat, Englewood, NJ, USA).

## Results

**Fig. 1:**
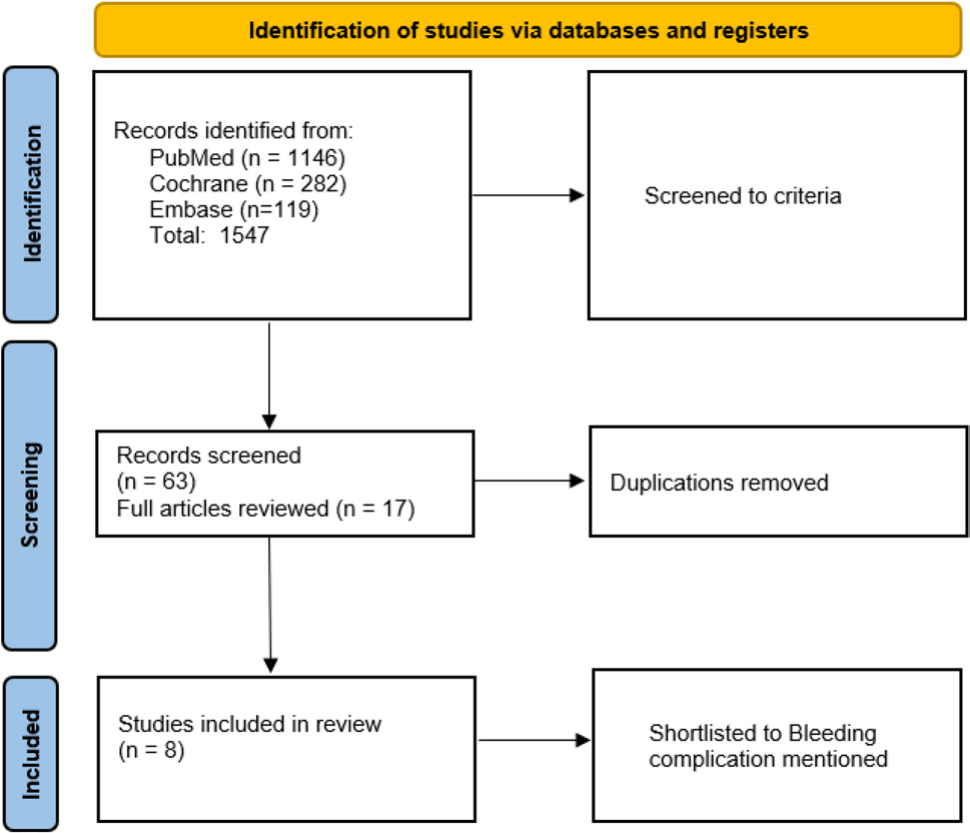
Study methodology and selection criteria using PRISMA (preferred reporting items for systematic reviews and meta-analyses)

The database search resulted in a total of 1547 articles. Following initial screening and removal of duplications, 8 articles were selected for full review and all 8 articles were deemed suitable to be included in the analysis (Fig. 1, Table 1). A total of 871 injection/aspiration procedures were included. Details of the different joints undergoing injection/aspiration and the rate of bleeding and other complications are shown in Table 2.

**Table 1:** Study demographics of the 8 included studies

Article by	Study method	Study type	Age	Sex (M:F) (%)	Needle size	Injection/Arthrocentesis	Knees (*n*)	Shoulder	Other joints	Follow-up
Conway	Cohort study	Prospective	Mean: 74	80:20	21G, 23G, 25G	Steroid and lidocaine injection or arthrocentesis	24	1*	Elbow- 1 MTP -1	4 weeks
Bashir	Case series	Prospective	Mean: 71	53:47	19G, 21G	Steroid injection	58	28**	0	4 weeks
Salvati	Cohort Study	Prospective	Mean: 65	53:47	19G	Arthrocentesis	15	**0**	0	1 week
Pandit	Cohort Study	Prospective	Mean: 72	100:0	Not Stated	Steroid and hyaluronate injection	91	56***	Elbow epicondyle-6 Hand Joints- 11 Wrist-5	1 month
Ahmed	Cohort study	Retrospective	Mean: 73	42:58	20-22G	Injection or arthrocentesis	Not stated	Not stated	Hip joint- < 3% of cases	24 h and 30 days
Thumbo	Cohort study	Prospective	Median: 74	Not stated	18F, 20F	Steroid injection	8	0	Elbow-1 Wrist- 2 Ankle- 2 First MTP- 2	4 weeks
Mian	Case series	Retrospective	Median: 77	66:34	Not Stated	Not stated	Not stated	Not stated	Not stated	1 month
Malige	Case series	Retrospective	Not stated	66:34	Not stated	Steroid and lidocaine injection	Nil	Nil	Hand and Wrist- 22	1–2 months

*Glenohumeral Joint

**Subacromial, Glenohumeral Joint

***Subacromial, Glenohumeral Joint

**Table 2:** Summary of complications encountered in the included studies with corresponding INR values

Author	Total procedures/patients	INR value	Timing of INR prior to procedure	Bleeding	Infection
Conway	27	Median 2.4 (2.1–2.6)	1 day	Nil	Nil
Bashir	86	Mean 2.77 (1.7—5.5)	Mean 15 days	Nil	Nil
Salvati	15	Median 2.7 (1.3–5)	3 days	1- slight bleeding on aspiration, knee, INR 3.8 1—frank bleeding on aspiration, knee, INR 5	Nil
Pandit	169	Mean 2.5 (1.1–4.8)	1 month	2—bruising, INR 2.1 and 2.4, procedure and site not stated	
Ahmed	456	Mean 2.7	24–48 h	1—early bleeding, INR 2.3, procedure and site not stated	1—joint infection, procedure and site not stated
Thumbo	15	Median 2.6 (1.5- 4.3)	Median 1.5 days (0–129)	Nil	Nil
Mian	81 patients	Median 2 (1.5–2.6)	Not stated	Nil	Nil
Malige	22 patients	Not tested	Not stated	Nil	Nil

Three studies reported cases of bleeding. Salvati et al. [13] reported that 2 patients had blood in the aspirate, one was mildly blood stained and the other had frank hemarthrosis. Both had pseudogout, INR values of 3.8 and 5, respectively and were also taking NSAIDS. None had a further bleeding event at review after one week. Pandit et al. [14] reported 2 cases of bruising at the injection site, with INR of 2.1 and 2.4 and one was also on aspirin. Ahmed et al. [15] reported a case with early clinically significant bleeding with an INR of 2.3 (Tables 2, 3, 4).

**Table 3:** Bleeding complications in patients with INR of more than 3

Author	Procedures/Patients	INR > 3	Bleeding complications
Conway	27	0	0
Bashir	86	Not stated	0
Salvati	15	6	1—slight bleeding on aspiration, knee, INR 3.8 1—frank bleeding on aspiration, knee, INR 5
Pandit	169	Not Stated	0
Ahmed	456	103	0
Thumbo	15	7	0
Mian	81 Patients	0	0
Malige	22 Patients	Not stated	0

**Table 4:** Risk of bleeding complications with concurrent use of antiplatelet agents and warfarin in the included studies

Author	Procedures/patients	Aspirin	Clopidogrel	NSAIDS	Bleeding complication
Conway	27	Not Stated	0	Not stated	0
Bashir	86	Not stated	Not stated	Not stated	0
Salvati	15	Not stated	Not stated	9	2 on NSAIDS 1—slight bleeding on aspiration, knee, INR 3.8 1—frank bleeding on aspiration, knee, INR 5
Pandit	169	14 patients on aspirin	Not stated	Not stated	0
Ahmed	456	196 procedures overall	Not specified	Not stated	0
Thumbo	15	Not stated	Not stated	Not stated	0
Mian	81 Patients	Not stated	Not stated	Not stated	0
Malige	22 Patients	Not stated	Not specified	Not stated	0

**Fig. 2:**
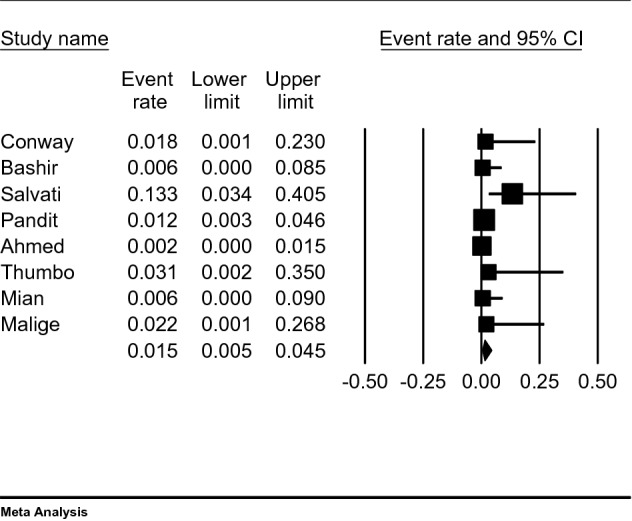
Meta-analysis of the included studies. Forrest plot showing estimated event rates and 95% CIs

Meta-analysis of all 8 included studies showed that the estimated bleeding complication rate associated with joint injection/aspiration was very low at 1.5% (95% CI 0.5–4.5%, Fig. 2) (heterogeneity: tau^2^ = 1.21; *I*^2^ = 50.43%; *Q* = 14.12; df = 7; *p* = 0.049) and for bleeding or infection 1.6% (0.6–4.4) (heterogeneity: tau^2^ = 0.99; *I*^2^ = 48.58%; *Q* = 13.61; df = 7; *p* = 0.059).

**Fig. 3:**
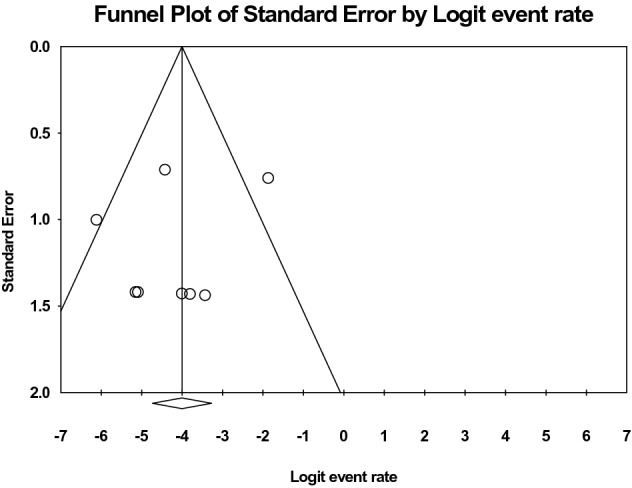
Funnel plot distribution of included studies

Funnel plot visual analysis did not show an obvious small study effect (Fig. 3). The outcomes of the critical appraisal of the included studies using MINORS criteria are summarized in Table 5. A clearly stated aim could be observed in all. However, unbiased assessment of outcomes was absent, as independent evaluators did not assess postoperative outcomes. The GRADE approach showed that the overall quality of evidence across the examined outcomes was “low” as the included studies were observational. The included studies had inconsistencies with regards to clinical heterogeneity but there was no significant variability in the reported results. There were no concerns for indirectness, publication bias and/or and imprecision.

**Table 5:** Methodological index for non-randomized studies (MINORS) tool to assess bias

Criteria	Conway	Bashir	Salvati	Pandit	Ahmed	Thumbo	Mian	Malige
A clearly stated aim	2	2	2	2	2	2	2	2
Inclusion of consecutive patients	2	2	2	2	2	2	2	2
Prospective collection of data	2	0	2	2	0	2	0	0
End points appropriate to the aim of study	2	2	2	1	2	2	2	2
Unbiased assessment of study endpoint	0	0	0	0	0	0	0	0
Follow-up appropriate to the aim of study	2	2	2	2	2	2	2	2
Loss to follow up, < 5%	0	0	0	0	0	0	0	2
Prospective collection of study size	0	0	0	0	0	0	0	0
Total	10	10	10	9	8	10	8	10

## Discussion

Our analysis shows that joint injection/aspiration may be performed safely in patients on warfarin with a low risk of bleeding or infection. Warfarin is the most used oral anticoagulant in the prophylaxis or treatment of atrial fibrillation, prosthetic heart valve replacement, DVT or pulmonary embolism [16]. There are an increasing number of, especially elderly patients on long-term warfarin [17]. However, warfarin has a narrow therapeutic window, and considerable inter-individual dose variations to achieve target anticoagulation level [18], which requires regular monitoring with INR testing. Under-anticoagulation can lead to life threatening thromboembolic events including cerebrovascular infarction, prosthetic cardiac valve thrombosis, DVT or pulmonary embolism, whilst over-coagulation may increase bleeding diathesis [19]. Conversely, bleeding is the most common adverse effect of warfarin [20–22], especially when undertaking any interventional procedures.

Some clinicians will stop the warfarin 5–7 days prior to the joint procedure whilst treating with low molecular weight heparin or unfractionated heparin and then restart the warfarin after the procedure. However, such an approach is time and health care resource consuming and may lead to a disturbance in warfarin control that may take several visits to re-establish the target therapeutic range [23, 24]. The alternative approach is to continue warfarin whilst carrying out the joint injection or aspiration. There is currently a lack of consensus about the management of warfarin for joint injections or aspiration. A survey of rheumatologists in the Yorkshire and Humber area of England, showed variable practice with some omitting warfarin, whilst others continued [25]. However, increasingly surgical interventions such as implantation of cardiac assisted devices [15], knee arthroplasty [26] and spinal interventions [23, 24] have reported minimal or no complications in patients who continued to take warfarin. These observations are in accord with our findings that support the overall safety of joint injections in patients on warfarin.

The INR at the time of the procedure is likely to influence the risk of bleeding. Bashir et al. carried-out injections regardless of the INR value, although their mean INR was 2.77, the highest being 5.5 and 87% of patients had an INR > 2 [27]. In the study by Ahmed et al. [15], 103 (22.5%) procedures were undertaken in patients with an INR > 3 and the highest INR was 7.81. In this study, only one patient with an INR of 2.3 had early periprocedural bleeding which did not involve the joint space [28]. In the study by Conway et al. [29] patients had a therapeutic INR value of more than 3 and they reported no complications.

Taking antiplatelet agents concurrently with warfarin may further increase the bleeding risk in patients undergoing joint injection or aspiration. Aspirin exerts its anti-platelet effects by irreversible inhibition of platelet cyclooxygenase (COX)-1, while NSAIDS bind reversibly and exerts a transient effect, but both increase the systemic bleeding tendency by impairing thromboxane-dependent platelet aggregation [30, 31]. Salvati et al. reported mildly blood-stained aspirate in one patient and frank hemarthrosis in another and both were taking NSAID for pseudogout, but also had elevated INR of 3.8 and 5, respectively [13]. In the study by Pandit et al., 2 patients developed bruising with an INR of 2.1 and 2.4, one of whom was also on aspirin [14]. In our selected studies, of 211 patients on warfarin, 3 were on concurrent aspirin and 2 had bleeding complications [13, 29, 30], whilst concurrent use of NSAIDS and Warfarin was reported in 10 patients, of whom 2 had bleeding complications [13, 28]. This suggests caution in patients on dual therapy.

The means of guiding needle entry into the joint during injection or aspiration may influence the risk of bleeding. Use of anatomical landmarks may require more needle adjustments to enter the required space, compared to ultrasound guidance (USG) and the latter may also identify and allow avoidance of large subcutaneous vessels. In the study by Bashir et al. orthopaedic surgeons carried out 72 injections using anatomical landmarks and interventional radiologists undertook 14 procedures under ultrasound guidance and neither group had a bleeding complication [28].

The type and depth of joint being injected may influence the risk of bleeding. Small joints such as of the hand and foot, more deeply seated joints such as the hip joint or joints with arthritic changes may make needle entry into the joint more challenging. The approach to access a specific joint space, may also influence the risk of bleeding. In the shoulder, multiple approaches may be used including the anterior or posterior for the glenohumeral joint and anterolateral or posterior approach for the subacromial space with a theoretically increased risk of damaging the cephalic vein in the anterior approach or the anterolateral shoulder vessels in the anterolateral approach to the sub-acromial space [32, 33]. In the knee the superolateral and superomedial, anterolateral and anteromedial approaches have reported different benefits and disadvantages [34]. Our included studies used a wide range of approaches and it was not possible to undertake further sub-analysis. For the shoulder, Bashir et al. [28] injected the subacromial and glenohumeral space using both a posterior/lateral approach and anterior/ posterior approach, whilst Ahmed et al. [15] performed shoulder joint injections in the glenohumeral and subacromial space and Conway et al. only injected the glenohumeral space. For the knee, Bashir et al. [28] used the superolateral and superomedial approach and Salvati et al. [13] used the lateral approach for aspiration. Malige et al. [35], performed procedures in hand and wrist, none of the patients on warfarin had any bleeding complications.

This study has certain limitations. Firstly, the studies were observational, with a lack of randomization. There was substantial heterogeneity with regards to the type and part of the joint injected (joint space or soft tissue) and size of needle used, although most procedures were undertaken for the knee and shoulder as expected in usual clinical practice. There was variation in the timing of INR assessment prior to the procedure, although most were within the expected range for their respective conditions. Some studies included joint soft tissue injections but most of them referred to joint space injections/aspirations. Nevertheless, our meta-analysis has allowed the pooling of a large number of cases to produce the most robust outcome assessment of the safety of joint injection/aspiration in patients on warfarin to guide clinicians and patients alike.

## Conclusion

This meta-analysis shows that it is relatively safe to perform joint injections or aspiration in patients on warfarin without routine prior testing of INR. Nevertheless, precautions such as identification and avoidance of any subcutaneous vessels, utilization of an approach that avoids any deep-seated vasculature and minimizes the number of attempts required to reach the intended part of the joint, as well as application of local pressure at the puncture site may further help to minimize any bleeding risks in this patient population.
